# Product Development of Natural Fibre-Composites for Various Applications: Design for Sustainability

**DOI:** 10.3390/polym14050920

**Published:** 2022-02-25

**Authors:** Muhammad Rizal Muhammad Asyraf, Agusril Syamsir, Nazirul Mubin Zahari, Abu Bakar Mohd Supian, Mohamad Ridzwan Ishak, Salit Mohd Sapuan, Shubham Sharma, Ahmad Rashedi, Muhammad Rizal Razman, Sharifah Zarina Syed Zakaria, Rushdan Ahmad Ilyas, Mohamad Zakir Abd Rashid

**Affiliations:** 1 Institute of Energy Infrastructure, Universiti Tenaga Nasional, Jalan IKRAM-UNITEN, Kajang 43000, Selangor, Malaysia; mohd.supian@uniten.edu.my; 2 Civil Engineering Department, Universiti Tenaga Nasional, Jalan IKRAM-UNITEN, Kajang 43000, Selangor, Malaysia; nazirul@uniten.edu.my; 3 Department of Aerospace Engineering, Faculty of Engineering, Universiti Putra Malaysia (UPM), Serdang 43400, Selangor, Malaysia; mohdridzwan@upm.edu.my; 4 Laboratory of Biocomposite Technology, Institute of Tropical Forestry and Forest Products (INTROP), Universiti Putra Malaysia (UPM), Serdang 43400, Selangor, Malaysia; sapuan@upm.edu.my; 5 Advanced Engineering Materials and Composites Research Centre (AEMC), Department of Mechanical and Manufacturing Engineering, Faculty of Engineering, Universiti Putra Malaysia (UPM), Serdang 43400, Selangor, Malaysia; 6 Department of Mechanical Engineering, IK Gujral Punjab Technical University, Kapurthala 144603, India; shubham543sharma@gmail.com; 7 School of Mechanical and Aerospace Engineering, Nanyang Technological University, Singapore 639798, Singapore; amma0002@e.ntu.edu.sg; 8 Research Centre for Sustainability Science and Governance (SGK), Institute for Environment and Development (LESTARI), Universiti Kebangsaan Malaysia (UKM), Bangi 43600, Selangor, Malaysia; mrizal@ukm.edu.my; 9 Research Centre for Environment, Economic and Social Sustainability (KASES), Institute for Environment and Development (LESTARI), Universiti Kebangsaan Malaysia (UKM), Bangi 43600, Selangor, Malaysia; szarina@ukm.edu.my; 10 School of Chemical and Energy Engineering, Faculty of Engineering, Universiti Teknologi Malaysia (UTM), Johor Bahru 81310, Johor, Malaysia; ahmadilyas@utm.my; 11 Centre for Advanced Composite Materials (CACM), Universiti Teknologi Malaysia (UTM), Johor Bahru 81310, Johor, Malaysia; 12 TNB Grid Division, Grid Solution Expertise (GSE), Bangunan Dua Sentral, No. 8, Jalan Tun Sambanthan, Kuala Lumpur 50470, Malaysia; zakir.rashid@tnb.com.my

**Keywords:** natural fibre-composites, conceptual design, product development, design for sustainability, sustainability development

## Abstract

New product development review article aims to consolidate the principles and current literature on design for sustainability to seek the field’s future direction. In this point of view, the design for sustainability methods can be established under the idea of sustainability in dimensions of ecology, economy and social pillars. Design for sustainability concept is implemented in concurrent engineering, including concept, embodiment and detail design processes. Integrating sustainability in engineering designs is crucial to producing greener products, system innovation, and services aligned with current market demand. Currently, many concurrent engineering studies related to natural fibre-reinforced polymer composites associated with sustainability enhance the application of design for sustainability techniques by professional designers. However, the current literature is scarce in bridging the design for sustainability concept with concurrent engineering during the design development stage, and these areas should be further developed. Several other future research directions, such as the need for aligning with principles and applications, along with exploring the relationships between the design for sustainability techniques and views of sustainability, are presented in this review paper.

## 1. Introduction

In the past few years, the development of various bio-based products with enhanced and high performance to cater to long-term environmental issues. This issue has raised concern among engineers and researchers due to the limited and scarcity of conventional resources on a global scale [1,2,3]. These resources, such as petroleum and coal, are very useful in developing numerous applications, including automotive, aerospace, energy and household-based products. According to Tiseo [4], the global production of petroleum-based plastics has grown significantly from 1.5 million tonnes to 367 million tonnes in 2020. Since the limitation of these conventional-based material resources has escalated the global demand, which contributes to a significant increase in the price value of the raw materials. This led the world society to shift their interest toward more environmentally friendly based materials such as natural fibres, biopolymers and biocomposites [5,6,7].

The natural fibre-composites have several demerits, such as poor interfacial bonding and exhibition of the hydrophilic property of natural fibres. The high abundance of hydroxyl groups makes the natural fibres hydrophilic, which lead to poor bonding with polymer matrices [8,9]. This issue could lead to composites’ poor mechanical, thermal, and physical properties. Thus, fibre modifications are using either chemical or physical treatments are proposed to resolve these issues [10]. Additionally, hybridizing of fibres with optimal orientation and lamination sequence is also proposed to enhance these properties [11,12]. Additive fillers such as MMT, CNT and nanocellulose are also a practical approach to improve technical properties of fibres such as thermal insulation and electrical conductivity besides the composite ‘s stiffness and strength [13,14,15,16]. In conjunction with these solutions, these approaches have to be embedded in the design process of the natural fibre-composite products to produce desired properties for specific functionality.

In conjunction with this issue, engineers are initially prepared to design green products using a concurrent engineering approach. A proper design is beneficial to developing a high-performance product to ensure durability and reliability. Generally, the overall concurrent engineering process includes concept generation, concept clarification, concept development and concept selection [17]. To satisfy the required design intent with better performance and environmentally friendly products, conceptual design is a vital stage in early product development with defined possible solutions. Specifically, four main areas were considered in the product design development process: idea generation, idea refinement, concept design development, and concept design selection. Inside these areas, various systematic approaches are available to be applied by designers and engineers [18].

A product design specification (PDS) is a critical aspect that a researcher or developer needs to cover to ensure the project maturity is in conjunction with the product’s requirement. Predominantly, around 32 elements are listed in product design specifications (PDS) for the conceptual design development process [19]. A design usually requires starting with a basic framework, including their primary design data, to lead the entire development process [20]. PDS is also necessary to achieve success for the product since it contains vital information about the product’s output. The boundary requirements must be reasonable in order to avoid predicting the outcome. Hence, all areas related and covered to the product have to be included to ensure customers’ fundamental aspects are fulfilled.

In the process, several systematic approaches are used to aid the engineers and designers to generate ideas for design concepts from problems and issues, such as Theory of Inventive Problem Solving (TRIZ) and brainstorming [21]. For instance, Cascini et al. [22] have implemented the TRIZ method in order to develop a conceptual design of sheet metal snips. They applied the TRIZ contradiction method to clarify and elaborate the idea generation for the new sheet metal snips conceptual design. Later, the final design concept of sheet metal snips is further enhanced using CAD software as design optimisation tool. Some studies also stated that the output from TRIZ contradiction method also can be refined further the generated solutions using morphological chart. A study led by Sapuan [23] has been incorporated morphological chart in their study to generate ideas for conceptual designs of polymer composites automotive pedals. The application of morphological chart was oriented according to principle of function analysis. In this method, a new concept designs were produced via the combination of multiple design features according to their product functions.

Other than that, the concept design selection stage is one of the crucial steps in finalising the concept design for product development. The available methods and tools of this stage applied in product development include Analytic Network Process (ANP) [24] and Technique for Order Preference by Similarity to Ideal Solution (TOPSIS) methods [25]. Both tools have their own specific advantages in providing essential solutions to decide the best design from the listing concept design produced. In specific, the multiple design attributes and design alternatives were analysed concurrently to fit the intended design specifications. Moreover, it also can be applied in the group decision-making process in conceptual design development. Generally, the natural fibre-composites have been widely used in various applications, including automotive parts [24], cross arm structures in the energy sector [26,27], insulation panels from coir fibre reinforced phenol formaldehyde biocomposite [28,29] and hybrid energy reed and rice straw fibres reinforced phenol formaldehyde biocomposite [30].

Thus, this review focuses on biocomposite product development by implementing the design of sustainability (DfS) in the concurrent engineering process. Generally, the product development covers those processes such as design concepts, material selection and manufacturing process selection in an integrated manner with the DfS concept to achieve sustainable green products and technology. Along with the design process, several concurrent engineering techniques were employed to ensure the design intends, characteristics of the design, and the selection of material were optimized before producing the final product. Furthermore, design concepts and materials can be combined in a computer system, namely, computer-aided drawing (CAD) and finite element analysis (FEA).

## 2. DfS: Implementing Design in Sustainability

**Figure 1:**
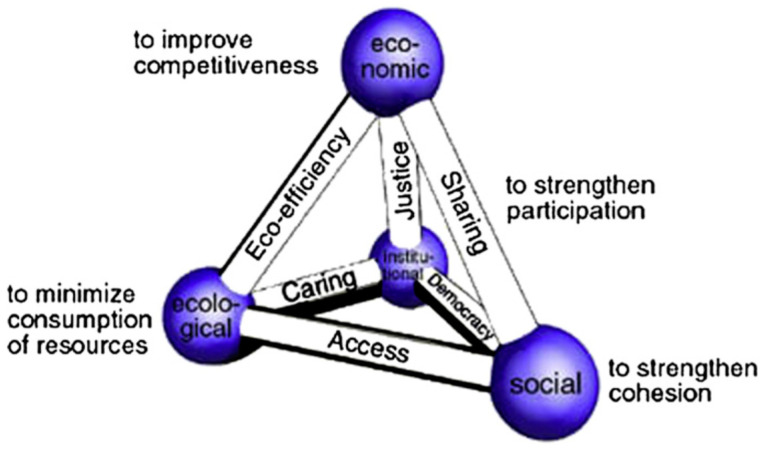
The Prism of Sustainability. Adapted with permission from Ref. [31]. Copyright 2010 Elsevier.

Generally, a correlation of sustainable consumption and production with four main pillars of sustainability is called a design for sustainability (DfS) [31]. Economics, ecological, institutional and social are those main key pillars adopted to ensure sustainable life quality, as shown in Figure 1 [32,33]. To practice sustainability in the view point of designers, they have to embed these four pillars to guarantee good governance of pertaining the resources up to producing the final product. Therefore, it is essential to entrench those constituents, including consumption and production views [34,35]. The use of the most appropriate technology, materials and production processes to achieve zero-carbon emissions and minimal non-renewable resource use whilst paying due attention to the impacts on human well-being (mental, physical and emotional) [3,36,37].

Environmental sustainability has been the primary purpose of many designers, especially engineers, which implement in the design process by considering sustainability as a possible contribution to the solution [38]. Fundamentally, DfS is a designing technique for physical objects, the built environment, and services to fulfil the principles of ecological sustainability [31]. However, the progress of design usually involves several factors in business competition, whereas eco-design is one of the essential components in developing green technology and products. Globally, many nations such as Japan and the United States of America are putting their efforts into creating energy-efficiency programs by driving green technology as their investment priority [39]. Meanwhile, the emerging global power nation, China, is firmed in order to achieve global leadership in green technologies [40]. For European Union (EU), many national initiatives have been initiated for green technology development, such as the Europe 2020 strategy, which narrowed on resources and information efficiency technologies and issued on the contribution of eco-design [41].

The idea of DfS is very distinct from eco-design. The eco-design focused on a method dealing majorly between economic and environmental effects from a life cycle analysis. This life cycle analysis is covered in terms of cost and impacts, such as life cycle costing and life cycle analysis (LCA). In conjunction with this matter, DfS is a technical approach that addresses all dimensions of sustainability, which narrowed into more total production and consumption of a system [42,43]. Product (service) and their function ability are usually categorised between production and consumption of the products.

In general, the amount of yearly purchases represents one of the essential components of the gross domestic products (GDP), whereas household consumption is frequently misunderstood with economic welfare (a measure of welfare) [44]. Though most of these expenses are not optional, around 90% of energy consumption for a product is determined during the design phase, although 90% of the life cycle wide energy consumption takes place during the use phase as mentioned by Tischner [45]. Thus, a product’s decision commonly comes from business managers, which signifies a product with higher profits instead of satisfying human needs.

Most short-lived goods have been widely applied for environmental and economic reasons since they are primarily produced compared to durable products. However, this issue has been debated among many environmentalists and researchers. Theulation of durable products has been c The extensive maintenance of durable goods is led to elevating the monetary and resources costs without providing additional welfare. The maintenance typically includes cleaning, repairing, upgrading or even renovating to conduct their functionality [46]. From the statement mentioned above, several requirements to innovate from the previous products to get rid of outdated and unsustainable products. This would add value for the product to achieve more sustainability toward better economic and environmental welfare.

## 3. Role of DfS

Design for Sustainability (DfS) usually addresses all dimensions of sustainability where it merges the influence of satisfier efficiency with the efficiencies of supply and products. However, in the engineering viewpoint, the focus is the opposite, whereby both disciplines are complementary. In this case, those involved stakeholders are commonly overlooked due to different mentalities and frequently do not recognize their mutual dependency on both disciplines [47]. For example, the DfS involved in natural fibre-composites focuses on replacing the existing synthetic material such as ceramics, glass, synthetic plastic and steel. By implementing natural fibres such as plant-lignocellulosic fibres, this new development of “green” products could add up the number of biocomposite products in the market [48,49]. Meanwhile, the DfS of general polymer composites focuses on recycling this material for a new product to fulfil the customers’ desires.

Ironically, the customer society typically commands over specific services and products from mass production based on their individualistic identities. As a solution, any organization would use the design and marketing efforts to cater to this issue, which often leads to pseudo-satisfiers (or even inhibitors) advertised. Here DfS comes as an alternative toward providing sustainable satisfiers and improving the effectiveness of satisfiers [31].

Designers should be considered eco-design during the development of natural fibre-composite products in their design proposals. In other words, eco-design is a component of DfS that employs the integration of environmental systems into product development [50]. In this case, it is essential to select appropriate and optimal natural fibre for composite product development to fulfil its design intends. Karana [51] noted that the material choice is highly dependent on the product’s predecessor material to ensure the new material is safe to be used. Nevertheless, this technique leads the product’s material selection to become limited. Generally, the material selection in natural fibre-composite product development is necessary to produce an innovative product. Taekema and Karana [52], materials are differentiated based on their properties, such as tensile strength, thermal conductivity, and the ability of materials to be processed and shaped. These properties are tallied with those factors that influenced the composite materials such as fibre type, fibre shape (granular, short fibre or continuous fibre, etc.), fibre arrangement, matrix type (thermoplastic and thermosetting) [53,54]. This kind of activity must be completed in the conceptual design stage. Thus, designers should overview these fundamental properties such as technical characteristics (strength and stiffness), formability properties and sensory behaviour to inspire and stimulate them to decide on a particular natural fibre-composite.

## 4. Practice and Implementing of Sustainable Design

This section focuses on implementing practice in sustainability concepts in the design development process. First, it is essential to gather the required procedures to develop an effective, sustainable design. Several practical aspects need to cover through product, process and system levels to attain sustainable design development [55,56,57,58]. In this context, the first principle that should incorporate in sustainable design development was to implement the fundamentals of employing materials and inputs from recyclable and non-hazardous. Next, the design process must consider applying renewable energy that does not affect the natural environment during its manufacturing process. In addition, the developed product designs are required to be reusable, recyclable or re-manufacturable besides expanding the design concepts of using fewer resources and applying easy-to-repair techniques.

The implementation steps of sustainable concept are varied based on the implementation difficulty level [59,60,61]. In conjunction with this statement, the first stage is developing work practice and maintenance or housekeeping step. It can be classified as a simple stage for effective inventory management, monitoring, and scheduling in all production operations. For instance, in producing a sustainable product, it is required to decrease loss from leaks and ensure all equipment’s appropriately maintained. The next step is process optimization. This stage involved planning and developing fabrication processes of sustainable products to minimize waste, conserve raw materials, and reuse waste materials. Additionally, within this step, the implementation of energy-efficient technologies promotes remarkable outputs, which support sustainable concepts. In particular, implementing cryogenic approach [62], dry cutting and minimum quantity lubrication [63], modelling and optimization approaches [20], and artificial intelligence methods [64]. The following step is raw material substitution. This step is intended to substitute hazardous materials and chemicals which a high possibility of environmental impact with sustainable materials (low health and environmental impact). In this case, Ilyas et al. [65] developed a smartphone holder from roselle fibre reinforced polymer biocomposites to replace the common synthetic materials made from PVC plastics. The outcome of this step is to cut down the environmental and health effects and avoid the regulatory costs associated with the storage and disposal of materials. Later, new technologies are the step that depends on employing more energy-efficient systems to improve the environmental impact performance since it has effective capabilities of saving heat and energy.

**Figure 2:**
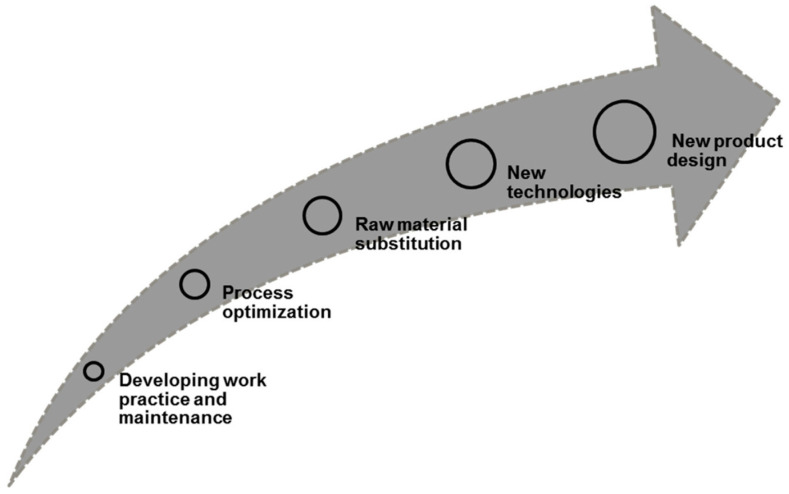
Implementation steps of sustainable design development. Adapted with permission from Ref. [66]. Copyright 2021 MDPI.

Nevertheless, applying new technology would require a huge capital investment problem. Lastly, another step which is the new product design considered the most challenging implementation step as it needs to transfer the whole system from the ground up to be more sustainable. These implementation steps are summarized in Figure 2.

Based on the previous literature, various studies conducted sustainable concepts on product development and linked them to sustainability aspects such as environmental and health concerns, energy consumption and waste management. Table 1 displays the recent technologies related to sustainable concepts.

## 5. Product Design and Concurrent Engineering

In the engineering field, product design is an important sub-field to have a successful commercial product. Products are usually defined as combinations of components that provide the functionality desired by customers. In general, a commercially successful product can be identified as an item that can produce an acceptable level of profit for the business entity. In this case, product design has to cover both the mechanical and industrial design criteria to ensure the product is well recognised by the users [75,76,77]. The development in materials goes hand in hand with improvements in manufacturing technology. Development of material and product design have been assisted by concurrent engineering knowledge, as has been expanded in detail earlier.

Concurrent engineering is a field that employs the knowledge to produce a good product development process. Product development is an essential step governed in concurrent engineering to produce a product within the time frame agreed between all the stakeholders involved [78,79,80]. These stakeholders include those manufacturers and suppliers in the market supply chain. It can rightly be claimed that CE is focused on milestones rather than the conventional focus on process.

Product development is a process that requires the total commitment of a product development group to progress a product right from a broad range design brief or customer requirement and details up to the manufacturing of a working prototype [81]. The process usually translates users’ desires into the physical product via product design and manufacturing processes. In the concurrent engineering viewpoint, product development can be defined as a combination of various functionality around the organization such as engineering designers, manufacturing engineers, suppliers, technical support personnel, administrative and sales personnel [82]. It safeguards good communication among them via advanced technology and information technology tools and even regular meetings. Product development has covered the product until it is marketed and other after-sale issues such as recycling, warranty, disposal, and maintenance [83].

Previous literature reports that various models were used in product development and design. In conjunction with this matter, three main core activities are usually carried out in a typical design process model: conceptual design, embodiment design, and detail design. Johnson et al. [84] propose the vital contributing factors to product design: sustainability and environment, aesthetics, science and technology, economics and investment climate, market and industrial design. The groundwork of product design is usually conducted by designers, such as design engineers and engineering designers [85]. Engineering designers cannot isolate themselves from others in the concurrent engineering field. Instead, they need to collaborate with other personnel to cater to all design problems in the early stage of the design process [86].

**Figure 3:**
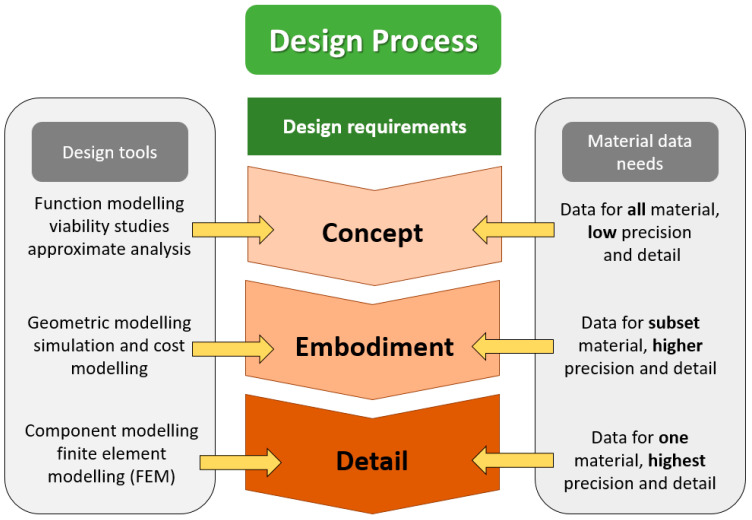
The design process in concurrent engineering.

In the conceptual design stage, the available resources are far lesser than required during the manufacturing stage. In this manner, it is still quite economical to make changes during this conceptual design stage. Thus, the application of concurrent engineering in product development would remarkably reduce costs by engaging other personnel early in the design process, whereby any changes done at this stage are desirable. A comprehensive design model composed of conceptual, embodiment and detail design is applied to conduct product design [53]. Those design inputs to this model such as solid modelling and finite element analysis were carried out to accomplish the expected product. Concurrently, the material selection must be conducted simultaneously to achieve the design input actions to consider the material in the beginning stage of the design process [87], as depicted in Figure 3.

## 6. Biocomposites as Sustainable Materials

**Figure 4:**
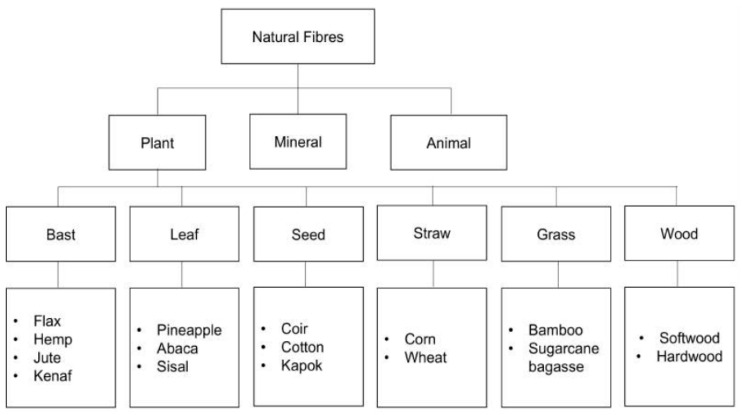
Schematic representations of natural fibres. Adapted with permission from Ref. [95]. Copyright 2021 MDPI.

**Figure 5:**
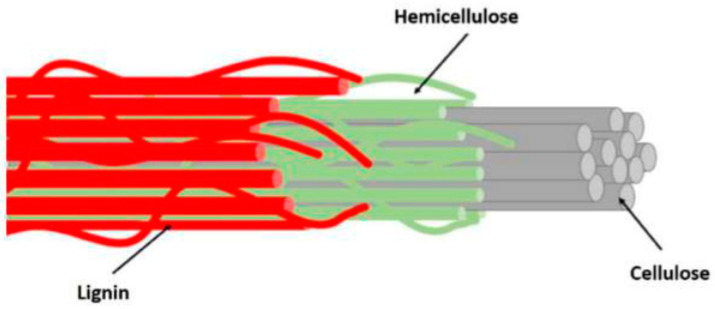
Schematic diagram of components of lignocellulosic fibre. Adapted with permission from Ref. [95]. Copyright 2021 MDPI.

A biocomposite material is usually clarified as a chemically distinct phase that is commonly attained from animal, mineral and plant wastes distributed within a continuous phase (polymer matrix) [88]. Commonly, the continuous phase which normally thermosetting and thermoplastic polymers, while the distributed phase is called the reinforcement. The classification of natural fibres can be seen in Figure 4. Here the natural fibres can be obtained from numerous resources; (1) plants such as oil palm, sugar palm, kenaf, pineapple leaf, banana pseudo-stem, coir, rice husk, wood, and bamboo, or even from (2) animal by-products including shells, skins and feathers. The plant-based fibres or lignocellulosic fibres are obtained from crop biomass waste such as pineapple leaf, sugar palm, oil palm empty fruit bunch and rice husk. These fibres are made up of cellulose components reinforced in a lignin matrix which exhibit high tensile strength [13,89] and thermal stability [10,90]. In general, the reinforcement can be laminate, particles, continuous or short fibres from plant cellulosic components (Figure 5). They are the second most abundant natural fibres for animal fibres after plant fibres. These fibres are made of proteins extracted from feathers, hair, silks and wools from animals’ parts and potentially reinforced in polymer composites [91]. The animal fibres are widely implemented in expensive textile uses. Those silk, wool and feather fibres have recently been applied as natural fibre-composites, especially for thermal insulation and acoustic applications [92]. The mineral fibres are the oldest natural fibres harvested from mineral rocks. Basalt fibre is the most common mineral fibre category used in polymer composites. Van de Velde et al. [93] discovered that basalt fibre-composites have significantly high tensile strength and modulus with lower density. This shows that this mineral fibre can potentially replace E-glass fibre as reinforcement in composites since it is resistant to extreme conditions. Generally, the mineral fibres-polymer composites are extensively electrical insulation and water filtration materials [94].

Variation of fibres, matrices, layering sequences, treatment, and fabrication techniques can be carried out to tally with the design intends [96,97,98]. The determination of composite compositions, with its chemical composition, synthesis and mechanical performance, is functioned to attain suitable biocomposite material to develop a product [95,99]. For instance, Ishak et al. [100] reported that sugar palm fibres are resistant to sea water and have excellent water barrier properties. Subsequently, Misri et al. [101] had developed a sugar palm fibre reinforced polymer composite boat due to its excellent mechanical and water-resistant performance. The fibres usually were arranged in many techniques such as multi-directional, randomly distribution of short or continuous fibres, bi-directional, unidirectional [102,103]. The choice of the matrix is extensive, covering a range of thermoplastics and thermosetting polymers. Biocomposites have been applied in various engineering applications, covering automotive, aerospace, civil, marine, etc. The application of biocomposites has risen in recent years because of improved awareness of product performance. Moreover, competition among industrial players in the global market for lightweight components has accelerated the use of biocomposites in many sectors [104,105].

## 7. Concurrent Engineering for Biocomposites

**Figure 6:**
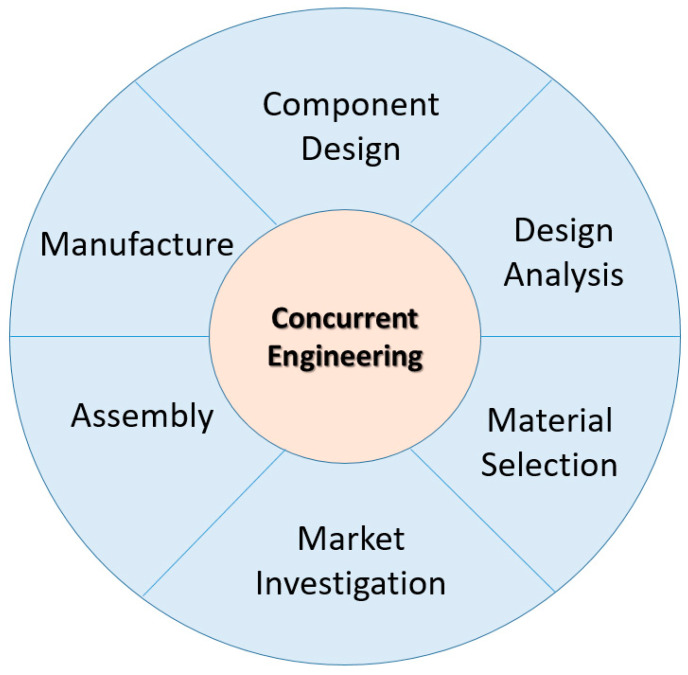
Concurrent engineering wheel.

In developing a new biocomposite product, it is necessary to ensure all aspects are considered in total, including concept generation, customer needs, product design specification (PDS), and detailed design to be manufactured [106]. In the detail design process, component design and process design were those elements covered in this stage. Strong [107] mentioned that component designs usually covered the sub-activities such as drawing or layout, constraints, analysis and material selection. Meanwhile, process designs include manufacturing method selection, sequencing, machine/tool selection, system layout, integration of system and manufacturing procedures. In other way round, Pugh [19] explained the concurrent engineering is not only relevant during the detail design stage but it also covered other processes of the total design including product design specification, market investigation and conceptual design. Even though there are two distinct philosophies in the total design process, both Strong [107] and Pugh [19] entail simultaneous consideration of the manufacturing process at the product design stage. For instance, the designer should embrace design features to facilitate ease of fabrication to reduce fabrication complexity if the component is to be manufactured using the hand lay-up process. This is due to the labour cost is a key cost element in the hand layup process. Figure 6 summarizes selected activities during the concurrent engineering process, also known as the CE wheel that may lead to a ‘wheel of fortune’.

## 8. Integrating DfS with Other Concurrent Engineering Techniques: Biocomposite Product Development

**Figure 7:**
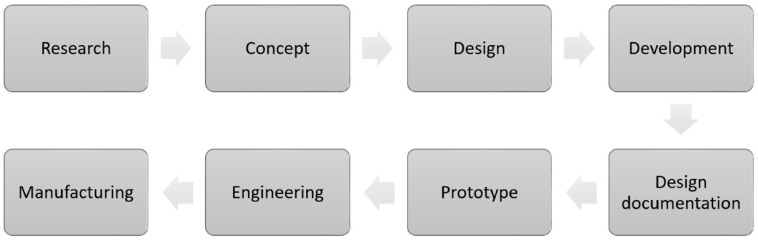
Biocomposite product development flow. Adapted with permission from Ref. [105]. Copyright 2021 MDPI.

Biocomposite product development involves three main stages, which are design development, selection of materials and prototype fabrications [108]. A specific design process flow is shown in Figure 7. Specifically, the product development started with the research activity to solve the problem statement as proposed in research gaps in the literature. At this point of view, the results or outcomes from the research were transformed into concept ideas before they developed into several conceptual designs [18,21]. Next, these conceptual designs were analysed with computer simulations to indicate the best design with actual conditions and functionality [109]. At this stage, all findings and design configurations were documented before being fabricated as a prototype. Finally, this prototype is tested to ensure its functionality and safety for users before mass production.

Several steps of the DfS technique are required to follow to fulfil the objective of sustainable products to design a biocomposite product. First, the DfS is obligated to design-wise by reviewing the background of the product with its function. Later, this step followed with the satisfaction that brings to the user. Commonly, the DfS approach exploits the Design for Excellent (DfX) to produce a sustainable product.

**Figure 8:**
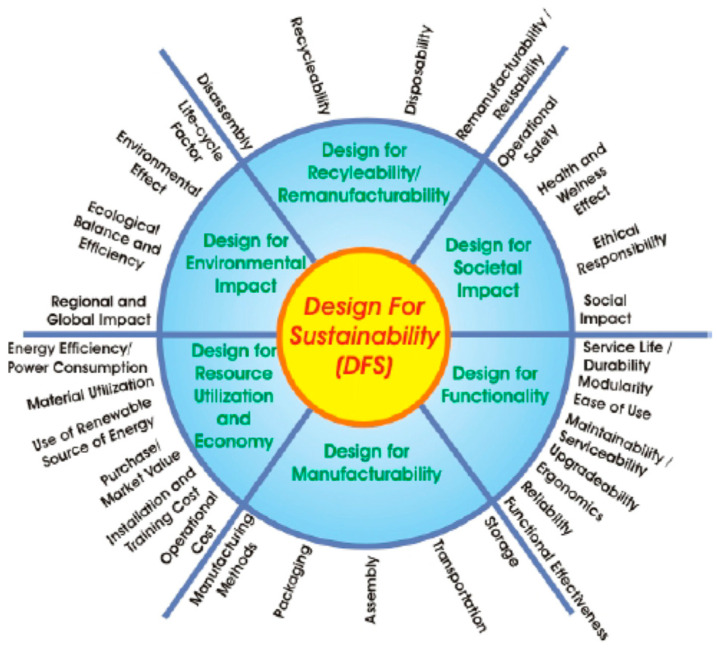
Elements within DfS. Adapted with permission from Ref. [47]. Copyright 2007 Dialnet.

Generally, DfX is a technical guideline applied when designing a biocomposite product for optimization based on a specific aspect of design [110]. The DfX implemented the concept of sustainability since the product implemented natural fibre-composites. The development of natural fibre-composites products required a practising designer to incorporate the sustainability concepts and ideas from the resources until producing final products. The fundamental element needed in the natural fibre-composites product development is the raw material obtained from renewable resources such as natural fibres or biopolymers. In this case, incorporating these constituents as building blocks of natural fibre-composites would reduce carbon emissions and adhere to minimal non-renewable resource use. Subsequently, this would increase the human well-being toward a sustainable environment [34]. This process adopts the analysis of its environmental impact from specific design attributes. These attributes include safety and biodegradability prospects to develop sustainable components/products. Jawahir et al. [47] had proposed a conceptual framework for DfS based on the DfX principles as displayed in Figure 8.

In product development, concurrent engineering is applied to optimize satisfactory human needs and sustainable products before the manufacturing process [79]. Specifically, the development of biocomposite products covers those components of sales trends and life cycle analysis. Raw material and production costs, consumer’s demands, and products’ production are those main components that need to be highlighted in these analyses [111,112]. The DfS usually is implemented with several concurrent engineering tools and techniques to design and conceptualise a specific sustainable product. Those concurrent engineering techniques include Extending the Search Space; Voice of Customer; Gallery method; Systematic Exploitation of Proven Ideas or Experience; Morphological Method; and Theory of Inventive Problem Solving (TRIZ). These tools and techniques are implemented in the conceptual design stage, which contains four essential sub-clusters (concept generation, selection, clarification and development) to fulfil the product’s design intends [18,21].

Later, to visualise and characterize the concept design produced, these concepts are integrated with computer software tools such as material selection tool [87], computer-aided drawing (CAD) [24] and finite element analysis (FEA) [20]. These computer tools aid the designers in further refining the design selection process before the fabrication of the prototype.

### 8.1. VOC

VOC or Voice of Customer is one of the concurrent engineering techniques implemented to create ideas for the design intends of the product. Generally, the VOC involved various aspects, including direct customers’ demands, surveys, focus group discussions, field reports, warranty data, interviews or discussions. From these aspects, the VOC data were extracted and later, they were incorporated in a product planning matrix, also known as quality function deployment (QFD) [113]. The QFD usually is generalized as customers’ requests and transforms these sorts of information into system plans to create customer requests and turn this information into systematic plans to produce products to meet those desires [113].

### 8.2. Systematic Exploitation of Proven Ideas or Experience

The systematic exploitation of experiences or proven ideas is another tool used by many designers to ignite the design intends before fabricating a prototype. These ideas or experience information are gathered through a series of discussions called brainstorming to physically analyse the current products. The brainstorming activities usually gathered and planned the generated ideas by mind mapping the problems and possible solutions, conceptualising the generated ideas, possible manufacturing processes and predicting the prototype’s characteristics [111]. In general, this technique occurs by analysing either the current product from the competitor’s or older version products from the previous model. Moreover, similar products available on the market also can be analysed via this technique based on the sub-functions of their function structures [114].

### 8.3. Morphological Chart

This concurrent engineering technique employs the usage of a chart in different arrangements, which helps the design process choose the new combinations of attributes or new sets of elements. The term “morphology” refers to understanding the form or shape of the material. Meanwhile, a “morphological chart” is signified as a systematic design technique to produce and examine the characteristics of the product which will be chosen [115,116]. In common practice, the chart is used for various options in terms of elements and components, which can be combined to become several expected solution ideas. In this case, the hybridization of these elements and components form multiple design features for the specific product functions.

### 8.4. Extending the Search Space

Extending the search space is usually implemented by designers by producing the “Why? Why? Why?” technique. This would aid the user to justify the search options by asking the root cause of the specific problems [117]. For instance, generally, the question would be, “Why do we need safety in composite products?”. The question will be followed with another why solution such as “Why the composite features needed to improve in certain aspects to ensure the product safety?”. This process is why the question will continue until the comprehensive conclusion statement is grasped. Nevertheless, this concurrent engineering technique is highly dependent on luck, and in turn, a brainstorming discussion has to be conducted frequently to generate solution answers for the final product features.

### 8.5. TRIZ

Theory of Inventive Problem Solving (TRIZ) is a problem-solving tool used in design development to generate solution ideas via inventive principles. This technique would cater to the problems that arise from specific problems, which converted to general problems [118]. The technique employed to remove the negative drawbacks during solution development and focused on the root cause of the problem [119,120,121]. In general, TRIZ can be divided into four main approaches: Contradiction Engineering with 40 Inventive Principles, Algorithms of Inventive Problem Solving (ARIZ), Su-field Modelling, and Prediction of Technology Trends [122]. Those techniques depend on the complexity level of the targeted problems. TRIZ aids to solve a problem systematically by identifying opportunities and innovation techniques to address the problem [123].

### 8.6. Multi-Criteria Decision Making (MCDM) Method

Many researchers usually apply Multi-criteria decision making (MCDM) to develop and select the optimised product design using numerical evaluation. The examples of MCDM tools to compute the design selection process are such as Analytic Hierarchy Process (AHP) [77], Analytic Network Process (ANP) [124], Technique for Order Preference by Similarity to Ideal Solution (TOPSIS) [125] and VIseKriterijumska Optimizacija I Kompromisno Resenje (VIKOR) [126]. Those tools are essential during the decision-making process based on multiple attributes with design alternatives that must be analysed simultaneously. Later, the proposed alternative designs will be matched the intended design specification and also be able to be applied in the group decision-making process. For instance, Hambali [79] developed a concept design for polymer composites automotive bumper beams using the AHP tool. From the proposed selection frameworks, the hybridization of AHP with another concurrent engineering tool would govern the ideal concept design with the best material and fabrication process specifically for the composite automotive bumper.

### 8.7. Gallery Method

Designers can use this technique to produce ideas by simultaneously displaying many generated concepts with a discussion. Typically, these concepts are visualised by sketched and taped on the designer’s design room wall to circulate each point of the ideas. Later, designers would suggest any improvements to the concept ideas or suddenly generate related ideas from this process [127].

## 9. Manufacturing Methods of Natural Fibre-Composites

Like other composite materials, biocomposites were made of natural fibre reinforced polymer composites that required specific fabrication techniques depending on the product applications. The natural fibre-composites possessed high specific strength and were good in biodegradability. Due to these reasons, biocomposites are considered high demand composite materials in the current markets. The global natural fibre-composites market size was valued at USD 4.46 billion in 2016 and is expected to grow to 11.8% from 2016 to 2024 [128]. To apply the biocomposites in many applications, especially structural applications, the material has to be guaranteed its optimal constituent elements and their suitable manufacturing process [129,130,131]. Variation of manufacturing techniques for natural fibre-composites is depended on production volume, product purpose, properties, shape and size [89,118]. The preliminary assessment to choose appropriate processing techniques depends on the size and shape of the composites.

Generally, the bulk manufacturing process of short fibre reinforced thermoplastic composites apply profile manufacturing by extrusion [132] and injection moulding [133]. For the fabrication process of low performance, continuous fibre reinforced polymer composites products, the hand and spray lay-up were commonly used because the raw material is cheaper [134]. These processes are considered open moulding techniques and relatively required non-professional fabricators [135]. Nevertheless, these open moulding techniques only can produce low fibre volume fractions and consume high volumes of void, which lead to less strength and high porosity [13,90,136]. For high-performance natural fibre-composites, the vacuum bagging technique is applied because the process emits less void between fibre-matrix by implementing pressure for consolidation [137]. Furthermore, autoclave vacuum bagging can produce higher performance and surface finish natural fibre-composites since it can add further external pressure to the bag [138].

Another manufacturing method that can be implemented for high-performance natural fibre-composites is compression moulding. Typically, this technique applied a hydraulic press between hot male and female moulds [9]. The moulds are usually heated by platens and pressured the composite laminate with certain pressure. The moulds are usually of steel to withstand the high closing forces. For thermosetting-based polymer composites, shaped open mould cavities were implemented with fibre reinforcement and resins. The preform may be assembled as a mould for the composite’s shape. The mould with the layering composite is pressed as closed, and the part is cured under pressure. Specifically, the natural fibre reinforced thermoplastic composites were usually manufactured using compression moulding via preheating a stack of pre-impregnated sheets separately from the mould tool [139,140]. This technique is also called stamping, which is commonly used to produce large glass fibre mat reinforced thermoplastic composite for automotive parts. Generally, the pre-impregnation process is considered tricky and timely. Thus, thin sheets of reinforcement and polymer can be ‘film stacked’. After the pre-impregnation stage, the preheating process is conducted in infrared lamps. As the thin-film stack softens, they are quickly placed in the mould tool and simultaneously shaped and cooled. Three main criteria that determined the process rate of this technique are heat transfer rates, the ability of the matrix to flow and accomplishing optimal impregnation and consolidation.

Resin transfer moulding (RTM) is another emerging manufacturing route that can produce high volume production for small-sized composite components of complex shape [141,142]. For this manufacturing technique, a mould is filled with dry fibres. Later, the polymer resin is transferred to the fabric layers before it is cured. While RTM is appropriate for relatively small components, the mould closure forces become excessive as component size increases.

Meanwhile, the natural fibre-composite products can also be fabricated by automated manufacturing methods such as filament winding [118,143], pultrusion [144] and robot-assisted fibre placement [145]. Furthermore, the machining process for composites also has been discussed in many previous works of literature [146,147,148]. Thus, biocomposites can be manufactured in many ways depending on the applicability, size and purposes.

## 10. Recent Development of Biocomposites Product Based on DfS

Currently, numerous new emerging environmental regulations have initiated the implementation of bio-based material products, especially in the conceptual design development stage. DfS is a concurrent engineering technique used to attain better environmentally friendly products and processes. Specifically, the term DfS is used to describe this block of activities and to those various levels of intervention that have the aforementioned variable dimension of changes required by the transition towards a sustainable society. This technique also aims to aid the researcher in developing a product or system that satisfies the well-being of environmental stakeholders, such as flora and fauna. To achieve the DfS objectives in conserving the environment, the DfS approach is conducted by balancing three vital components: profit, human wellness, and the environment. The main element proposed in the DfS approach is to ensure a design implement the application of renewable energy alongside opting for solutions that enable the reduction of energy usage [43]. In this case, natural fibre and biopolymer film are those renewable resources to develop various products to support this aim. This section explains the summary of the current progress of applications of concurrent engineering design to develop environmentally sustainable products in terms of current research works.

In concurrent with this view, the development of bio-based products has gained the intention of engineers and designers since it has lesser production cost and their promising factors such as lightweight property and the resources are widely available [149,150]. As a result, mechanical engineers have initiated various bio-based products, designers and researchers. In general, the development of the conceptual design of a product implements chronological order of presentation, such as identifying the current problem to generate concept design, developing concept designs and selecting the final concept designs. However, these techniques are limited to product development and can be applied to system innovation and services [151].

In the generating of green products in concurrent engineering, it should be clearly mentioned the future ability of the product in terms of environments (less greenhouse emission), economic (reduced cost for users) and social (better equity in the ability to use the product) issues. However, the analysis would be straightforward if the researchers and designers made some distinction in the model’s description. Hence, an element such as “environmental”, “economic”, and “social” criteria are essential in analytical frameworks to generate innovative ideas, improvements the current state of the product and criteria for assessment. However, it was observed that in several models, the idea generation criteria and the assessment criteria are the same. Therefore, a joint analysis was performed. Table 2 displayed the recent development and advancement of bio-based product development using several concurrent engineering models and techniques.

Mansor et al. [152] mentioned the development of a parking brake lever from kenaf fibre polymer composites using the integration of TRIZ, morphological chart and AHP. This work aims to develop an environmentally friendly product for the automotive interior part to replace the existing steel-based parking brake lever. Three criteria in DfS are implemented to reduce the steel-based products’ current cost and ensure the proposed product is a par in terms of structural strength for functionality and safety performance, besides promoting greener material-based products. In this work also, they use the TRIZ contradiction matrix as a problem-solving tool to extend the main sustainability idea [18,21]. Later, the morphological chart was implemented to refine the generated inventive solution produced from TRIZ to develop the concept design of the parking brake lever. Lastly, the authors formulated the Analytic Hierarchy Process (AHP) for the design selection process based on the established product development specifications using pair-wise comparison.

Also, Mansor et al. [154] also used the TRIZ contradiction matrix and morphological chart to produce the conceptual design of car spoiler using kenaf fibre-composites. In addition, Ishak et al. [156] has developed natural car front hood from fibre –aluminium laminate composites by using the TRIZ if-then-but technique along with the TRIZ contradiction matrix. Another research work led by Mazani et al. [111] has developed a kenaf fibre reinforced unsaturated polyester composites shoe rack using the integration of the TRIZ-morphological chart-weighted objective method. Initially, they generate the idea from brainstorming and mind mapping process to link the current problem with the sustainable issue before detailing it in the TRIZ method. The brainstorming and mind mapping techniques aid them to view the current situations and issues holistically before coming to a solution.

**Figure 9:**
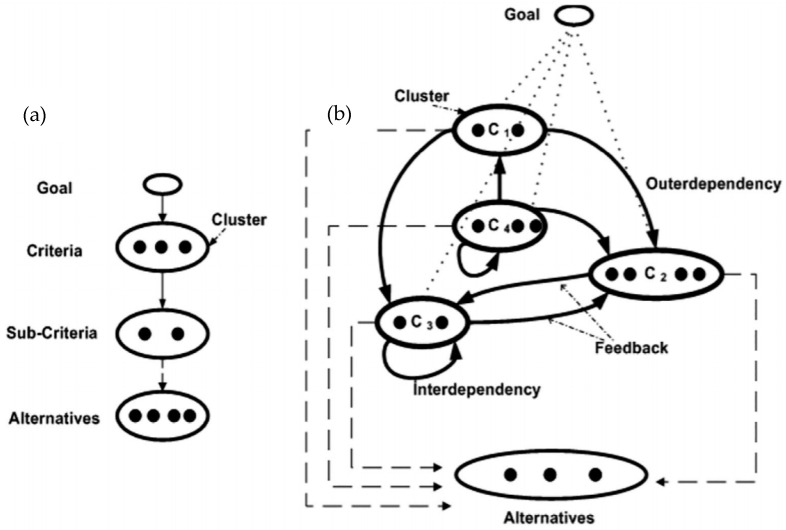
The general concept of AHP (**a**) and ANP (**b**) in the design selection process. Adapted with permission from Ref. [118]. Copyright 2020 Wiley.

Apart from that, Azammi et al. [24] and Asyraf et al. [118] implement the same problem identifier tool (TRIZ contradiction matrix) and refine generated ideas (morphological chart). However, both research works differentiated their works from Mansor et al. [152] to produce automobile rubber engine composite and portable fire extinguishers, respectively, by implementing Analytic Network Process (ANP). The ANP is a generalisation of AHP, which depends on the feedback in the decision structure. In this case, the AHP is a technique applied to govern the general decisions by dividing them into multi-level hierarchy structures (composed of aim, criteria and alternatives). Meanwhile, ANP is a tool to develop decisions from complex inter-relationships between each decision level and their attributes. Figure 9 shows the schematic diagram to elaborate the general concept of AHP and ANP tools.

Lastly, other concurrent engineering methods were conducted in various ways, such as integration of TRIZ-BOS-Morphological Chart-AHP [155], TRIZ-Biomimetics-VIKOR [81], brainstorming-Pugh evaluation method [157] and Voice of Customers-TOPSIS [153]. These integrated techniques developed optimized products by aiding them with finite element analysis such as fluent and structural analyses [20].

## 11. Influence of Ecological, Social, Economic and Institutional Factors in Development of Natural Fibre-Composites Products

Natural fibres have shown that the material has high economic value in agricultural upstream and commercialized downstream products. For instance, kenaf fibre commodity is projected to reach US $854 million by 2025 [158]. In this case, kenaf fibres can be directly used as raw material for pulp and papers, increasing the demand for natural fibre. Apart from kenaf, other natural fibres such as pineapple, banana, sugar palm, roselle, etc., also show the significant growth of demand in domestic and international trades to boost the socioeconomic status of the global populations [159,160,161]. Consequently, the phenomenon would eradicate mass poverty, deprivation, and underdevelopment in local communities [36,162]. However, even though the economic cycle of natural fibres seems promising, several issues were encountered by farmers, including low production output as the main current problem [163]. For instance, the turnover rate for kenaf crops is wide-ranging from 5–58 years based on financial analysis from Mohammed [164]. Thus, government agencies should take action by urging the creation of a production chain ranging from crop cultivation up to industry stakeholders to solve the problem [165].

**Figure 10:**
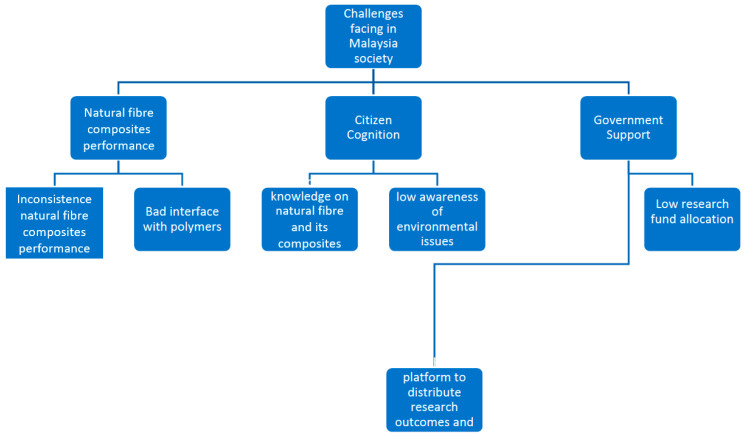
The challenges faced by natural fibre reinforced polymer composites.

Generally, an excellent supply-demand chain is a good indicator of the economic value of natural fibre commodities. However, the natural fibre reinforced polymer composites seems to have less value in commercial stages due to issues summarized in Figure 10. The natural fibres can be reinforced in both synthetic and bio-based polymers [89,130,131,166]. The issues are related to natural fibre-composites performance besides society recognition and government support. Further clarification on these issues were detailed in subsequent paragraphs.

The mechanical performance of natural fibre is highly dependent on chemical components in the fibre. In this case, the high degrees of inconsistent chemical components of natural fibres would generate different tensile strengths and modulus between individual fibres [167,168]. This led manufacturers to walk away from choosing natural fibres as a substitution for synthetic fibres in engineering applications. On the other hand, the synthetic fibres are identical in all aspects, which become favourable for manufacturers. Apart from that, the hydrophilic behaviour of natural fibres was discovered to be incompatible with hydrophobic polymers. This hydrophilic nature of natural fibres also leads to higher water absorption uptake, which accelerates the degradation process and causes may induce failure in composites’ functionality and structural integrity [90,169]. These two statements showed that these factors demotivate manufacturers to apply natural fibre-composites for advanced products, which may cause sudden failures or malfunctions. In the end, the failures of the product may lead to the loss higher amount of money and/or valuable lives.

Most reports describe that the surface treatment of natural fibre can significantly enhance fibre-reinforced polymer composites’ mechanical performance. Nevertheless, the issues arise as fibre treatment would incur the cost and production cycle-time [170]. This has become a selection dilemma for the industry to use natural fibres as reinforcement in composites. Luckily, the awareness of environmental conservation is improved from day to day [105]. Therefore, the implementation of natural fibres has become a selling point used by companies to improve their reputation to grasp customer attention. However, industrial stakeholders should take the options and apply the natural fibre reinforcement, as this is a future path in applying green material development for sustainable technology. As a resolution, industrial funding, collaborations, and feedback are the key elements needed to develop natural fibre-composites products, especially at the commercialization stage, aligning with the product’s demand criteria.

Even though the statement mentioned above solutions are highlighted, the awareness among society toward environmental issues is still below the threshold to promote green technology. For example, Malaysia generated 4.0 million tons of solid waste in 2019 [158]. It can be seen that plastic disposal percentages have been inclined dramatically due to the COVID-19 pandemic. This has been due to the usage of personal protective equipment (PPE) such as gloves, gaunt, face masks and shoe covers by medical workers to examine and treat patients. In terms of psychological viewpoint, people tend not to buy products that they don’t know about. Thus, education and public awareness on natural fibre-composites have to promote to improve the public’s knowledge by indicating the green materials’ performance and potential for various usages. Subsequently, it will reduce the saturated municipal solid waste situations. Currently, research carried out by Ramachandralu [171] has developed surgical clothing made of bamboo fibres to reduce the dependence on conventional plastic. Thus, researchers, scientists and engineers have to work together to promote the application of other natural fibres such as kenaf, sugar palm and pineapple as disposal masks and other PPE products.

Nowadays, the implementation of natural fibre-composites is the perfect time to expose for the public. This would entail full support from the government agencies and local universities. However, the deficiency of education dissemination of research achievements through innovations remain among the research society and fail to be shared with the public. Thus, the government should form comprehensive platforms among researchers and the public to deliver ideas, information, findings and innovations to all levels of citizens. Online newspapers, public campaigns, social media and community services are those effective channels for entrenching achievements by scientists related to natural fibre-composite [172]. Unfortunately, several middle-income nations, such as Malaysia, have a lesser amount of research funding in terms of annual expenses compared to high-income nations. The lack of funding would decline the research advancements, limiting recruitment and sharing at international conferences.

In prospects, the advances of natural fibre-composites toward sustainable development have been significantly progressing either locally or internationally. Thus, this review paper focuses on explaining an overview of the knowledge of biocomposite product development, the applicability of biocomposite for sustainable development and their challenges. This would allow researchers to shift their interests and plans for future studies on the natural fibre-composites.

## 12. Conclusions

This review paper established an informative summary of biocomposites which covered the broad spectrum of biocomposite product development involving the design of sustainability and other concurrent engineering techniques. Generally, the biocomposites are formed by natural fibres (plants, animals and minerals) reinforced in the polymer matrix. The plant-based lignocellulosic fibres were usually implemented in biocomposites due to their abundant resources, cheap cost, and remarkable mechanical properties for engineering applications. Sugar palm, jute, pineapple leaf, and roselle are fibres applied in many engineering and structural applications. However, several improvements of natural fibres should be conducted to enhance the performance of composites, such as fibre treatments, composite configurations, and additive materials. These aspects should be considered in the design stage of natural fibre-composite products. The appropriate product design of biocomposites is compulsory to boost the intended properties of the composite products and their materials toward optimized strength and functionality. The idea of implementing natural fibre-composites aligned with the DfS because it caters to four main pillars of sustainability during the procurement of the raw materials, design process, manufacturing products and functionality for end-users. The DfS would be an umbrella for product development, and subsequently, an optimization of ecological view, institutional policy, commercial economics and social benefits can be grasped concurrently. To optimise functionality and mechanical performance, engineering design methods such as brainstorming, TRIZ voice of customers (VOCs), and morphological charts are crucial. These techniques could outline users’ issues and solve them via the product’s functionality. These reviews elaborate on the current biocomposites product implements DfS and concurrent engineering techniques, which aids the reader to understand the process of biocomposite product developments further.

## Figures and Tables

**Table 1:** Sustainable technology in conjunction with its application.

Ref.	Sustainable Technology	Application
[67]	Application of various coolant pressures	Advance the machinability of Inconel 718 and Waspalloy
[68,69,70]	Implementing of MQL-nano-fluid	Improving the machinability of Inconel 718 and Ti-6Al-4V to improve tool wear, surface quality and power consumption
[71]	Implementing biodegradable oils with minimum quantity lubrication (MQL)	Accomplish sustainable machining (Inconel 718)
[72]	Hybridizing both techniques of MQL and cryogenic	Achieve environmentally efficient machining even in difficult-to-cut materials
[73]	Applying of 6R approach with the waste management process	Enhancing the construction waste recycling
[74]	Conducting vegetable oil with MQL	Attain the sustainable machining by ADI

**Table 2:** Recent developments and advancements of bio-based products using DfS in concurrent engineering approach.

Product	Final Concept Design	Bio-Based Material	Concurrent Engineering Techniques			No. of Concept Designs	Application	Ref.
			Problem Identifier Tool	Refine Problem Identifier Tool	Selection Concept Design Tool			
Parking brake lever	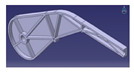	Kenaf fibre polymer composite	TRIZ (Contra-diction Matrix)	Morpho-logical Chart	Analytic Hierarchy Process (AHP)	5	Auto-motive	[152]
Automobile engine rubber composite	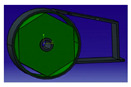	Kenaf fibre polymer composite	TRIZ (Contra-diction Matrix)	Morpho-logical Chart	Analytic Network Process (ANP)	4	Auto-motive	[24]
Automotive bumper beam	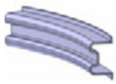	Hybrid natural fibre polymer composite	Voice of customer	Finite Element Analysis	TOPSIS	8	Auto-motive	[153]
Car spoiler	-	Kenaf fibre polymer composite	TRIZ (Contra-diction Matrix)	-	Morpho-logical Chart	1	Auto-motive	[154]
Side door impact beam	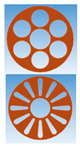	Natural fibre-composite	TRIZ + Biomi-metics	Finite Element Analysis	VIKOR	8	Auto-motive	[81]
Anti-roll bar	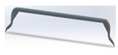	Hybrid natural-carbon fibre reinforced composite	TRIZ-Blue Ocean Strategy (BOS) four-action frame-work	Morphological Chart	AHP	42	Auto-motive	[155]
Car front hood	-	Natural fibre-aluminium laminate	TRIZ (If-Then-But Method)	TRIZ (Contra-diction matrix)	-	1	Auto-motive	[156]
Fire extinguisher	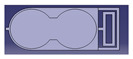	Hybrid natural fibre polymer composite	TRIZ (Contra-diction matrix)	Morpho-logical Chart	ANP	4	Build-ing safety	[118]
Shoe rack	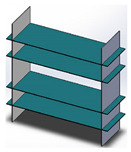	Kenaf fibre reinforced UPE composite	Brain-storming + Mind-mapping + TRIZ	Morpho-logical Chart	Weighted Objectives Method	3	House-hold	[111]
Portable laptop table	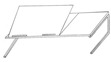	Kenaf fibre polymer composite	Brain-storming	-	Pugh evaluation method	9	House-hold	[157]

## Data Availability

All information and data are available within the articles.
